# Alterations of Gut Microbiome in Patients with Colorectal Advanced Adenoma by Metagenomic Analyses

**DOI:** 10.5152/tjg.2024.24294

**Published:** 2024-11-01

**Authors:** Jingyuan Xiang, Ningli Chai, Longsong Li, Xinwei Hao, Enqiang Linghu

**Affiliations:** 1 Department of Gastroenterology, the First Medical Center of Chinese PLA General Hospital, Beijing, China; 2 Medical School of Chinese People’s Liberation Army, Beijing, China

**Keywords:** Advanced adenoma, colorectal cancer, diagnostic model, gut microbiota, shotgun metagenomic sequencing

## Abstract

**Background/Aims:**

Colorectal cancer (CRC) is one of the deadliest cancers worldwide, mostly arising from adenomatous polyps. Mounting evidence has demonstrated that changes in the gut microbiome play key roles in CRC progression, while quite few studies focused on the altered microbiota architecture of advanced adenoma (AA), a crucial precancerous stage of CRC. Thus, we aimed to investigate the microbial profiles of AA patients.

**Materials and Methods:**

Fecal samples were collected from 26 AA patients and 26 age- and sex-matched normal controls (NC), and analyzed by shotgun metagenomic sequencing.

**Results:**

Gut microbial dysbiosis was observed in AA patients with lower alpha diversity. Advanced adenoma was characterized by an increased *Bacillota/Bacteroidota* ratio and higher *Pseudomonadota* levels compared to normal individuals. Linear discriminant analysis effect size (LEfSe) analysis was performed and identified 14 microbiota with significantly different abundance levels between AA and NC groups. Functional analysis revealed that tryptophan metabolism was upregulated in AA. Correspondingly, the expressions of gut microbes implicated in tryptophan metabolism also changed, including *Akkermansia muciniphila, Bacteroides ovatus, Clostridium sporogenes*, *and Limosilactobacillus reuteri. *The microbial network suggested that AA exhibited decreased correlation complexity, with *Escherichia coli* and *Enterobacteriaceae unclassified* harboring the strongest connectivity. A diagnostic model consisting of 3 microbial species was established based on random forest, yielding an area under the curve (AUC) of 0.799.

**Conclusion:**

Our study profiled the alterations of the gut microbiome in AA patients, which may enrich the knowledge of microbial signatures along with colorectal tumorigenesis and provide promising biomarkers for AA diagnosis.

**Main Points**

- Colorectal advanced adenoma is a crucial precancerous stage of colorectal cancer (CRC), while limited studies have focused on the gut microbial alterations in patients with advanced adenoma.
- By shotgun metagenomic sequencing, patients with advanced adenoma showed a characteristic gut microbiome shift compared to normal individuals.
- Microbial network analysis revealed that advanced adenoma exhibited a decreased correlation complexity.
- The diagnostic model based on microbial species could achieve considerable efficiency in the prediction of advanced adenoma.

## Introduction

Colorectal cancer (CRC) is the third most common cancer worldwide and the second leading cause of tumor-related deaths.^1^ Recently, the incidence of early-onset CRC among individuals younger than 50 years old has been continuously increasing, posing a threat to public health.^2^ It is well known that CRC initiation and progression are favored by the complex interactions of genetic and environmental factors. Hereditary CRC is estimated to account for 2%-5% of all cases, which suggests the critical role of environmental factors in colorectal tumorigenesis.^3^

Human intestinal tract is a vast ecosystem. Trillions of microorganisms (bacteria, viruses, fungi, and archaea) inhabit the mucosal epithelium, encoding 100 times more genes than the host’s own genome.^4^ Accumulating evidence has revealed that the gut microbiome is one of the most important environmental factors implicated in CRC development by producing secondary metabolites, triggering oncogenic signaling, and modulating immune microenvironment.^5^ In a 2019 study, Sobhani et al^6^ transplanted the fecal microbiota from CRC patients into germ-free mice, causing local mucosal inflammation and host DNA methylation, and subsequently promoting intestinal tumor formation. Microbial dysbiosis has been recognized as a hallmark of CRC, characterized by enrichment of procarcinogenic bacterial strains, such as *Fusobacterium nucleatum*, *Bacteroides fragilis,* and *Prevotella*. In contrast, various beneficial probiotics, including *Clostridium*, *Roseburia, *and *Faecalibacterium*, were found to be downregulated in CRC patients compared to healthy populations.^7^ Due to the characteristic composition of the gut microbiome in CRC, the potential value of certain microbial species as diagnostic biomarkers has been highlighted and clinically evaluated.^8^

Most CRC cases evolve in a multistep sequence termed as “adenoma-carcinoma,” and colorectal advanced adenoma (AA) is a crucial precancerous stage of CRC.^3^ Compared with non-advanced adenomas (NAA), AA lesions harbor more malignancy and appear to be the last benign stop before CRC. Till now, a number of studies have reported the global microbiota shifts during CRC progression,^9,10^ while there is still lack of trials mainly focusing on AA. Furthermore, the majority of prior research adopted 16S ribosomal RNA (rRNA) for microbiota identification, which would induce significant variance and provide limited taxonomic coverage (genus or above). Therefore, our study aimed to investigate the alterations of the gut microbiome in patients with AA using shotgun metagenomic analysis, thus contributing to a deep understanding of this pathological condition.

## Materials and Methods

### Patient Inclusion and Sample Collection

Our study was carried out at the First Medical Center of Chinese PLA General Hospital from February 2023 to October 2023. Patients with colorectal AAs were enrolled, and all the lesions were removed under endoscopy. Advanced adenoma was defined as those simple adenomas ≥10 mm, with villous histology or high-grade dysplasia (HGD).^11^ Final diagnosis was confirmed based on the post-operation histopathological results. Healthy individuals, who received colonoscopy for physical examination, were recruited as normal controls (NC). All the participants were local residents from the Beijing region.

The detailed exclusion criteria were as follows: (i) use of antibiotics, proton pump inhibitors, or probiotics within the past 3 months; (ii) history of inflammatory bowel disease (IBD), irritable bowel syndrome (IBS), or any primary cancers; (iii) organ failure or inability to endure colonoscopy; and (iv) pregnancy. Fecal samples were collected before bowel preparation and quickly stored at −80°C until DNA extraction. The study was approved by the Chinese PLA General Hospital’s ethics committee (approval no. S2022-082-01, date: February 24, 2022). Written informed consent was obtained from all subjects and the entire procedure was performed in accordance with the Declaration of Helsinki.

### DNA Extraction and Shotgun Metagenome Sequencing

Fecal sample DNA was extracted according to the manufacturer’s instructions provided by the E.Z.N.A.® Stool DNA Kit (D4015-02, Omega, Inc., Norcross, Georgia, USA) and dissolved in 100 µL sterile water for further analysis. The purity and concentration of the DNA extract were assessed with a NanoDrop Microvolume Spectrophotometer (Thermo Fisher Scientific, Waltham, MA, USA). The quality of DNA was evaluated by 1.5% agarose gel electrophoresis. Sequencing libraries were constructed using the TruSeq Nano DNA LT Library Preparation Kit (FC-121-4001). The gene library quality was confirmed with an Agilent 4200 (Agilent Technologies, Santa Clara, California, USA). We performed 2 × 150 bp paired-end sequencing on the NovaSeq 6000 platform with PE150 mode (Illumina, San Diego, California, USA).

### Quality Control

The raw data were processed to obtain valid reads. Briefly, adapters were removed from the sequencing reads using cutadapt v1.9, and low-quality reads were trimmed by fqtrim v0.94 using a sliding-window algorithm. In addition, the reads mapping to the human genome reference (hg38 database) were also discarded to avoid host contamination. Remaining reads were recognized as valid reads and adopted for the following gene prediction and annotation.

### Taxonomic Annotation

The quality-filtered reads were assembled to construct the metagenome for each sample with IDBA-UD. All coding regions (CDS) of metagenomic contigs were predicted by MetaGeneMark v3.26, and those contigs with a length shorter than 100 bp were eliminated. Coding region sequences of all samples were clustered by CD-HIT v4.6.1 software to obtain unigenes with a threshold of 95% sequence identity and 90% coverage. The unigene abundance profile was calculated based on the number of aligned reads. Subsequently, the lowest common ancestor taxonomy of unigenes was obtained by aligning them against the National Center for Biotechnology Information (NCBI) non-redundant (NR) database by DIAMOND v0.7.12 for taxonomic annotations.

### Bioinformatics and Statistical Analysis

Continuous variables were compared using a *t*-test, and categorical variables were compared using the Fisher’s exact test. A *P* value <.05 was considered statistically significant. Shannon and Chao1 indices (α-diversity) were measured to describe species evenness and richness. To examine the between-group discrepancy in microbial composition, principal coordinate analysis (PCoA, β-diversity) was conducted using Bray-Curtis distance with ggplot 2 package in R software (version 3.3.2). The linear discriminant analysis (LDA) effect size (LEfSe) was applied to determine the influential microbiota most likely to represent the differences between AA and NC groups. The threshold was set at LDA<3 and *P*<.05.

Microbial co-occurrence networks in AA and NC groups were established based on SparCC correlation analysis, respectively. Species with significantly different abundance (top 50) were selected and evaluated (correlation coefficient> |0.2| and *P*<.05). Differentially enriched pathways were identified by integrating unigenes into the Kyoto Encyclopedia of Genes and Genomes (KEGG) database. The prediction model for early detection of AA was constructed using a random forest classifier.

## Results

### Clinical Information of the Study Cohorts

**Figure 1.**
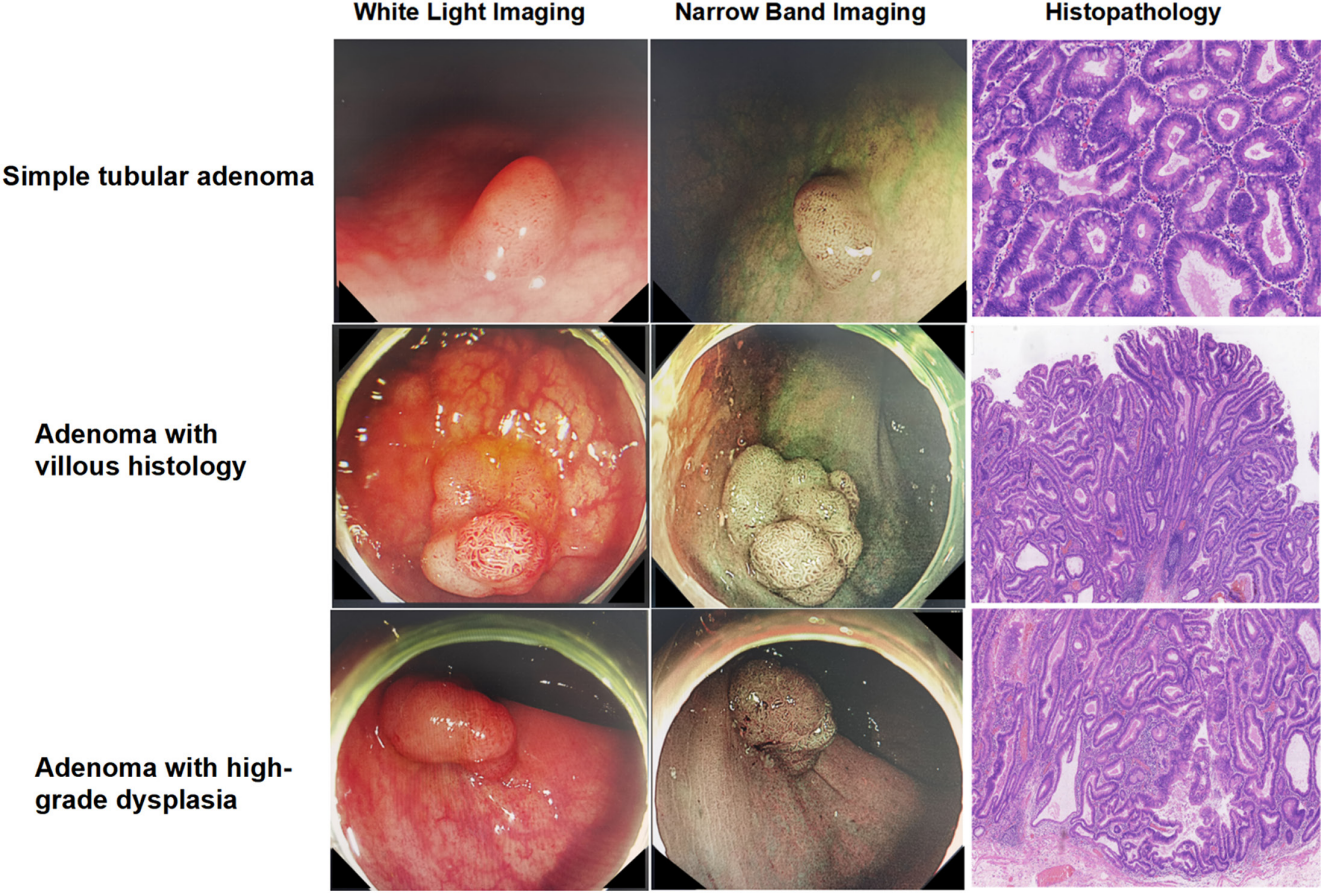
Endoscopic presence (WLI and NBI mode) and corresponding histopathological findings of colorectal advanced adenoma.

From February 2023 to October 2023, a total of 52 individuals were enrolled in the study, comprising 26 AA patients and 26 age- and sex-matched normal controls (NC). In the AA group, 5 patients were identified with tubular adenoma with a size >10 mm, 6 patients with villous histology, and 15 with HGD (Figure 1). The BMI and smoking status of the AA and NC groups were comparable (*P*>.05). The baseline data are demonstrated in Table 1. All participants were Han Chinese and lived in the Beijing region.

### Overall Analysis of Microbial Communities in Advanced Adenoma and Normal Control Group

**Figure 2.**
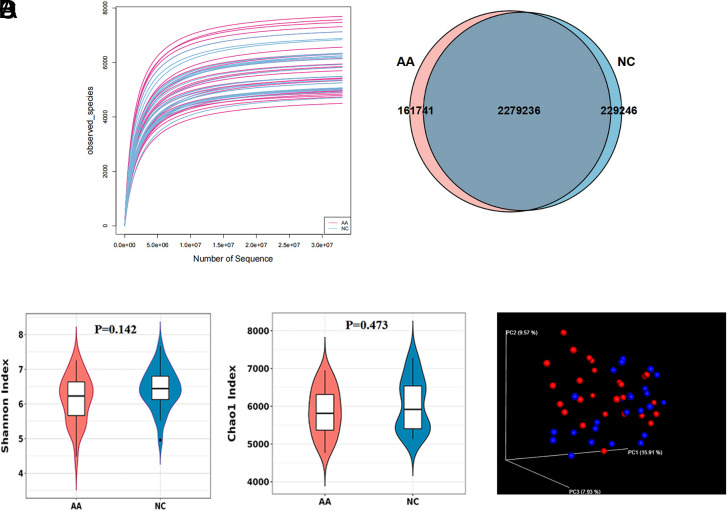
Fecal microbiome structure in AA and NC individuals. (A) Refraction curve based on the observed species. (B) Venn diagram showing the unique and shared genes in AA and NC groups. (C) Alpha diversity measured by the Shannon and Chao1 index. (D) Principal coordinate analysis (β-diversity) using Bray–Curtis distance.

After the data filtering process, 2 670 223 genes were obtained from 52 fecal samples by shotgun metagenomic sequencing. The refraction curve indicated a sufficient sequencing depth for microbiome analysis (Figure 2A). Venn diagram showed that there were 161 741 unique genes in the AA group and 229 246 unique genes in the NC group (Figure 2B).

Shannon and Chao1 index (alpha diversity) were used to evaluate the overall microbiota characteristics in terms of richness and evenness. Despite no statistical significance, the alpha diversity of the AA group tended to be lower than that of the NC group (Figure 2C, Shannon index, *P* = .142; Chao1 index, *P* = .473). Principal coordinate analysis (beta diversity) calculated by Bray–Curtis distance was performed to display the microbial community variations between the 2 groups. As shown in Figure 2D, we observed a marginal separation of different samples (ANOSIM, *R* = 0.07, *P* = .01), indicating a mild shift of the gut microbiome in patients with AA.

### Changes of Microbial Tax Between Advanced Adenoma and Normal Control Groups

To investigate the changes in taxonomic composition between AA patients and control subjects, the relative abundance of gut microbiota in different groups was evaluated and compared at the phylum, genus, and species levels, respectively.

**Figure 3.**
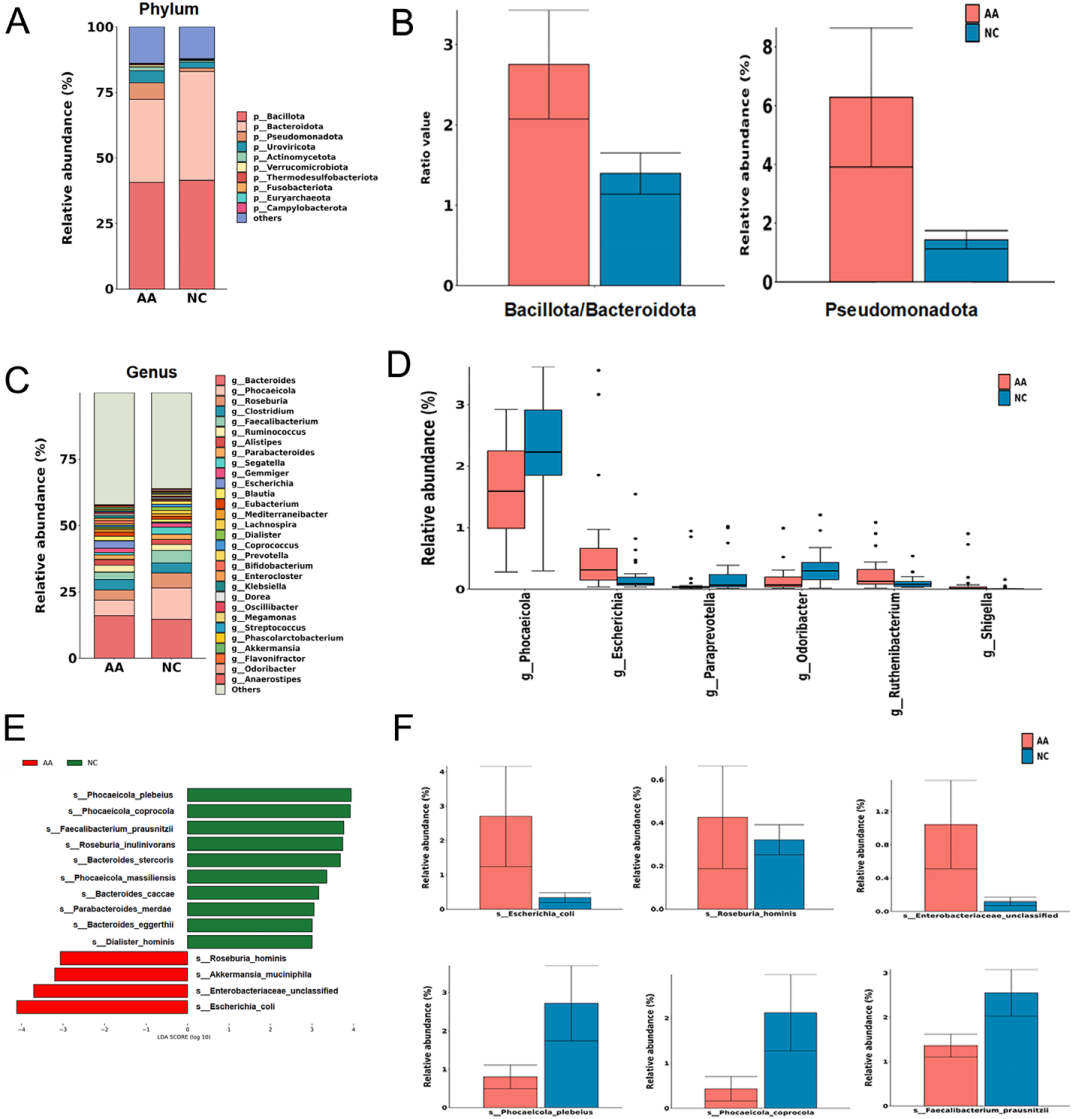
Gut microbiota signatures of AA and NC groups at different taxonomic levels. (A) Relative abundance of microbes (top 10) at the phylum level. (B) Increased Pseudomonadota levels and Bacillota/Bacteroidota ratio were exhibited in AA patients. (C) Relative abundance of microbes (top 30) at the genus level. (D) Significantly altered genus between 2 groups. (E) Most differentially enriched species identified by LEfSe analysis (LDA>3). Red bars indicated AA-enriched species, and green bars indicated NC-enriched species. (F) Bar plot of representative AA-enriched species, Escherichia coli, Roseburia hominis, and Enterobacteriaceae unclassified; and AA-depleted species, Phocaeicola plebeius, Phocaeicola coprocola, and Faecalibacterium prausnitzii in all samples.

At the phylum level, AA and NC groups were mainly composed of *Bacillota*, *Bacteroidota*, *Pseudomonadota*, *Uroviricota,* and *Actinomycetota*, which accounted for over 80% of the entire microbiota (Figure 3A). Notably, AA was featured with higher *Pseudomonadota* levels (*P*<.01) and an increased *Bacillota/Bacteroidota* ratio (AA vs. NC, 2.75 vs. 1.40, *P* = .26, Figure 3B).

At the genus level, *Phocaeicola* was obviously enriched in the NC group followed by *Paraprevotella* and *Odoribacter* (Figure 3C and 3D). In contrast, *Escherichia*, *Ruthenibacterium,* and *Shigella* were found to be more abundant in the AA group.

**Supplementary Figure 1.**
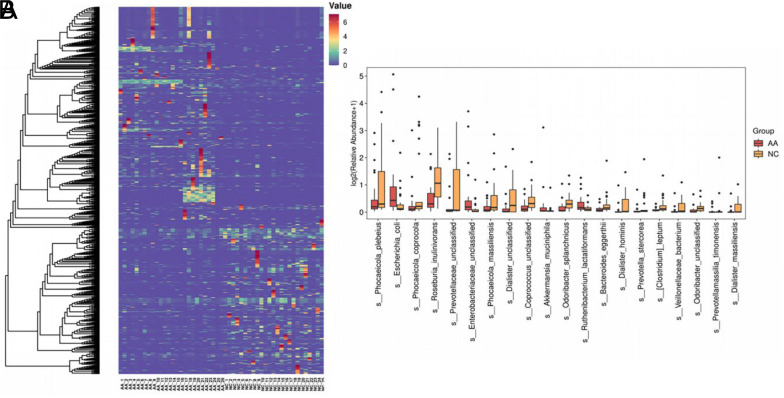
Microbial profiles of AA patients and NC individuals at the species level. (A) Heatmap showed a clear difference of microbial composition between two groups. (B) Differentially expressed microbiota species (top 20) in AA.

According to the microbial community profiles, 904 species were distinct between AA patients and controls (Supplementary Figure 1A). The top 20 species in average abundance are shown in Supplementary Figure 1B. LefSe analysis was then performed to identify the most differentially expressed species with an LDA score>3 (Figure 3E). We found that the levels of *Escherichia coli*, *Enterobacteriaceae unclassified*, *Roseburia hominis,* and *Akkermansia muciniphila* were enriched in the AA group, whereas 10 species were reduced in AA individuals, including *Phocaeicola plebeius*, *Phocaeicola coprocola*, *Faecalibacterium prausnitzii*, *Roseburia inulinivorans*, *Bacteroides stercoris*, *Phocaeicola_massiliensis*, *Bacteroides caccae*, *Parabacteroides merdae*, *Bacteroides eggerthii,* and *Dialister hominis *(Figure 3F).

### Functional Alterations of Gut Microbes in Advanced Adenoma Group

Altered gene abundance could reflect changing functional patterns. To investigate the characteristic pathways of AA, related genes were aligned to the KEGG database and compared based on their abundance with an e-value cutoff of 1e−5.

**Figure 4.**
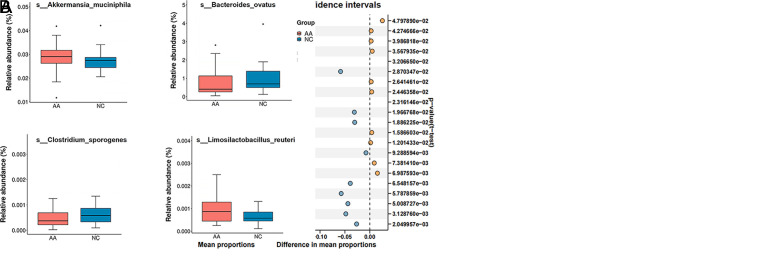
Functional analysis based on KEGG. (A) Significantly different pathways between AA and NC groups. (B) Representative microbial species involved in tryptophan metabolism.

Totally, 21 distinct pathways were identified between AA and NC samples (Figure 4A). Multiple pathways were elevated in the NC group including the biosynthesis of cofactors, biosynthesis of amino acids, and cysteine and methionine metabolism. Among the altered functional pathways, we found that the degradation of aromatic compounds and tryptophan metabolism were more abundant in the AA group. Tryptophan is an aromatic amino acid and its metabolites exhibit influential effects on CRC development. Correspondingly, microbial species involved in tryptophan metabolism were also differentially expressed between AA and NC groups (Figure 4B), such as *Akkermansia muciniphila, Bacteroides ovatus, Clostridium sporogenes, and Limosilactobacillus reuteri*. Thus, aberrant tryptophan metabolism might be an early event of colorectal dysplasia.

### Analysis of Microbial Ecological Networks in Advanced Adenoma and Normal Control Individuals

**Figure 5.**
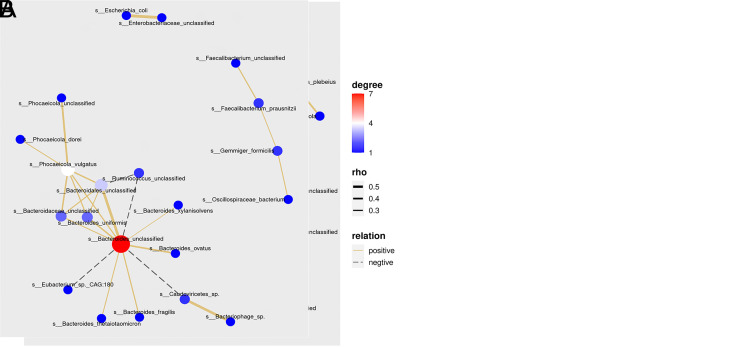
Species-level co-occurrence networks in NC (A) and AA group (B). Correlation coefficients among gut microbes were represented by edge width. Relative abundance of each microbe was represented by node color.

The stability of the gut ecosystem relies on microbiota cooperation and competition. To explore the complex interactions among microbial species in AA and NC groups, co-occurrence networks were established with SparCC. On the whole, AA patients displayed a reduced complexity of the microbial network with 21 nodes and 24 edges compared to the healthy subjects with 25 nodes and 32 edges (Figure 5).

Our results demonstrated *Bacteroides unclassified* was the major species contributing the most connections for both groups. Especially in the AA group, *Bacteroides unclassified* harbored positive correlations with 8 species (*Phocaeicola vulgatus*, *Bacteroidales unclassified*, *Bacteroides uniformis*, *Bacteroides xylanisolvens*, *Bacteroidaceae unclassified*, *Bacteroides ovatus*, *Bacteroides fragilis*, *Bacteroides_thetaiotaomicron*) and negative correlations with 3 species (*Caudoviricetes sp.*, *Ruminococcus unclassified*, *Eubacterium sp. CAG:180*). Moreover, the correlation of *Bacteroides unclassified* with *Bacteroidales unclassified* was found to be one of the strongest associations (rho = 0.4379, *P* = .0099), which implied the crucial role of *Bacteroides *as a network hub.

The decreased species network in AA might be due to lower microbial diversity, which indicated an impaired gut ecosystem. More probiotics in the NC group tended to establish tight connections, such as the *Roseburia* genus (*Roseburia intestinalis*, *Roseburia inulinivorans,* and *Roseburia unclassifie*d), *Lachnospira eligens,* and *Faecalibacterium prausnitzii.* By contrast, *Escherichia coli*, a typical pathogenic microbe, harbored the strongest inter-group connection with *Enterobacteriaceae unclassified* in the AA group (roh = 0.478, *P* = .0099). This correlation was not observed in the NC group, which suggested *Escherichia coli* might be the key pathogenic driver for AA.

### Microbiota-Based Diagnostic Model for AA

**Figure 6.**
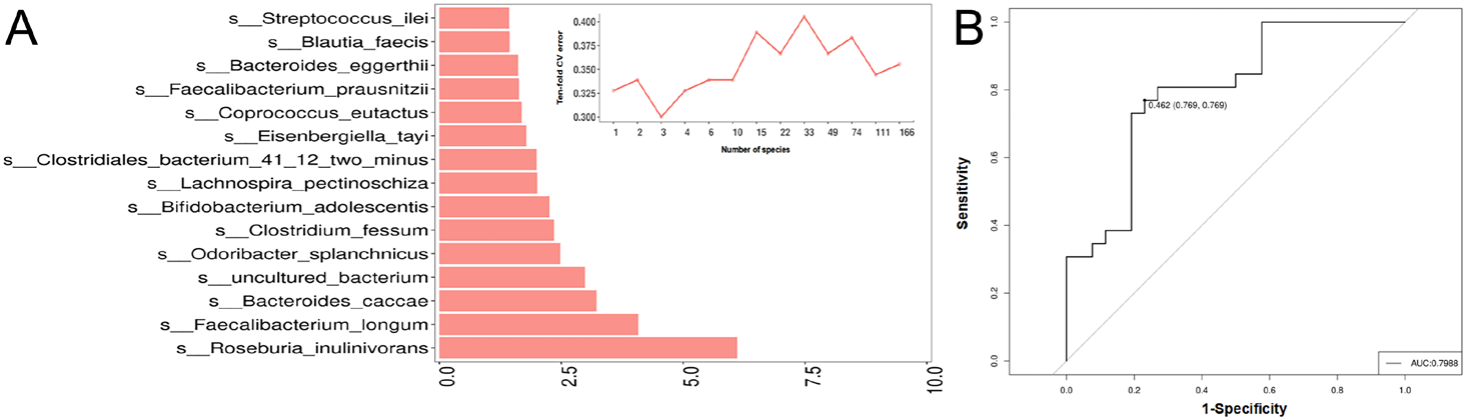
Establishment of microbiota-based diagnostic model for AA. (A) Ranking importance of microbial species by the mean decreased accuracy. The inset chart shows the cross-validation (CV) errors according to numbers of selected species. (B) AUC values of the diagnostic panel using 3 microbial species.

Next, we sought to evaluate the potential value of gut microbes as prediction biomarkers for AA. Using random forest, the importance of all the different microbiota between the 2 groups was ranked based on mean decreased accuracy. After 10-fold cross-validation, 3 species were finally selected to construct the diagnostic panel, including *Roseburia inulinivorans*, *Faecalibacterium longum,* and *Bacteroides caccae*, which were depleted in AA patients (Figure 6A). The 3-microbiota model exhibited an AUROC of 0.799; the sensitivity and specificity were both 0.769 (Figure 6B).

## Discussion

Here, we performed shotgun metagenomic analysis of fecal samples to unveil the gut microbiome alterations in AA patients. Microbial traits were investigated in terms of taxonomic compositions, functional pathways, and co-occurrence networks. In the meantime, a microbiota-based prediction panel for AA was developed using a random forest model.

Gut dysbiosis plays vital roles in human diseases including CRC. Previous research has screened that CRC is accompanied by specific changes in the microbiota community.^5^ For instance, the levels of *Gemella*, *Peptostreptococcus,* and *Parvimonas* were found to be positively correlated with CRC.^12^ Han et al showed that CRC patients were abundant in *Shigella*, a Gram-negative enteropathogen that could induce colon mucosa damage and promote tumor development with Shiga-like toxin.^13^ As a precancerous lesion of CRC, AA is the transitional status from normal colonic epithelium to malignant tumors. In light of this, AA should include a unique gut microbiome different from healthy populations or patients with CRC and NAA. Several published pieces of literature introduced the microbial features in AA, while the number still remained quite few and the conclusions seemed inconsistent across different studies.^9,14^

In our trial, the overall microbiota community of the 2 groups demonstrated subtle separation, and we identified a series of microbiota with distinct expression in AA patients. Notably, the *Bacillota/Bacteroidota* (formerly *Firmicutes/Bacteroidetes*, F/B) ratio was higher in the AA group, which is mainly caused by the reduction of *Bacteroidota *levels. *Bacillota* and *Bacteroidota* are 2 predominant phyla in the gut. The disorder of the *Bacillota/Bacteroidota* ratio has a close relationship with obesity, IBD, and benign prostate hyperplasia.^15^ The link between the *Bacillota/Bacteroidota* ratio and CRC was unclear. In recent studies, the ratio was found to be a potential risk factor for breast cancer and exhibited 3 times lower levels in patients.^16^

According to the LefSe analysis, 4 AA-enriched bacteria were screened, including *Escherichia coli*, *Enterobacteriaceae unclassified*, *Akkermansia muciniphila,* and *Roseburia hominis*. *Escherichia coli* is a representative CRC-promoting bacterium. It has been proven that *Escherichia coli* strains with pks islands can exploit virulence factors and induce DNA breaks in colon cells, thus contributing to CRC progression.^17^ In AA subjects, we found the level of *Escherichia coli *was increased 7.95-fold compared to that of healthy controls. The results of our microbial network analysis also demonstrated that *Escherichia coli *played an important role in AA pathogenesis.

Interestingly, we noticed that AA patients were also abundant in 2 probiotics, *Akkermansia muciniphila* and *Roseburia hominis*. *Akkermansia muciniphila* accounts for 3%-5% of the entire microbial species in the adult colon and confers influential benefits to the host immune system.^18^ Perturbation of *Akkermansia muciniphila* is linked with multiple diseases, such as diabetes, liver steatosis, and atherosclerosis.^19^ In mouse models, Jiang et al found that acetyltransferase from *Akkermansia muciniphila* could promote the cellular secretion of heat-shock protein 70 and the immune response of CD8+ cytotoxic T lymphocytes, thus inhibiting colorectal tumorigenesis.^20^ *Roseburia hominis* was found to be involved in gut health by producing butyrate.^21^ A decrease of butyrate-producing bacteria has been previously reported in CRC patients, exhibiting a reverse changing trend compared to AA.^22^ This discrepancy might be attributed to the precancerous nature of AA, and the elevations of the above 2 probiotic microbes could be a kind of adaptive response by the human host.^23^

Among the AA-diminished species, *Faecalibacterium prausnitzii* is one of the most widely studied with anti-tumorigenic and anti-proliferative properties. The depletion of *Faecalibacterium prausnitzii* was commonly identified in IBD cases,^24^ which share similar pathogenesis with colorectal AA. Moreover, *Bacteroides stercoris*, a short-chain-fatty-acid (SCFA) producing microbe, was found to be downregulated in AA. Short-chain-fatty-acid serves as beneficial agents in maintaining the gut homeostasis. Insufficiency of SCFAs would result in a pro-inflammatory microenvironment and lead to CRC progression.^25^ However, Lee et al noted that *Bacteroides stercoris* was more abundant in patients with tubular adenomas,^23^ which was different from our present findings. We suspected this may be partially due to the complexity and diversity of microbial analysis, which can be largely affected by individual factors such as age, diet habits, and antibiotic use. Moreover, only simple tubular adenomas>1 cm were included in Lee’s trial. The heterogeneity in sample composition could cause totally opposite conclusions. *Phocaeicola* was a remarkably enriched microbial genus in control subjects with increased *Phocaeicola plebeius*, *Phocaeicola coprocola,* and *Phocaeicola massiliensis*. Of note, *Phocaeicola vulgatus* harbored the second most connections in the AA group following the species of *Bacteroides unclassified*. Thus, the *Phocaeicola *genus may be one of the influential microorganisms tightly implicated in AA formation.

Our functional analysis revealed that the tryptophan metabolism pathway was up-regulated in the AA group. Tryptophan is an essential amino acid and has a relationship with various diseases, including CRC.^26^ As a tryptophan metabolite, kynurenine could result in immune tolerance and tumor development.^27^ On the contrary, derived from tryptophan as well, indolic metabolites demonstrate a tumor-suppressing nature by prompting cell apoptosis and enhancing antioxidant activity.^26^ In the present study, a series of gut microbiota differentially expressed in AA was implicated in tryptophan metabolism. For instance, *Limosilactobacillus reuteri *genomes are enriched with key enzymes, which are necessary to transform tryptophan into indolic metabolites.^28^ In a 2023 study, Zhang et al reported that *Akkermansia muciniphila *had an inhibitory effect on tryptophan metabolism via the AhR/β-catenin signaling pathway to counter CRC progression.^29^ Apart from tryptophan metabolism, the AA group was also more abundant with the biosynthesis of unsaturated fatty acids. Dysregulated lipid metabolism is an emerging hallmark of cancers due to rapid cell growth and increased energy demand.^30^ However, metabolic alterations triggered by host-microbe interactions appear to be crucial and complex along tumor development. Thus, further integrated analysis of the gut microbiome and metabolome is indispensable to investigate the exact role of metabolic reprogramming in AA.

With the advance in endoscopic techniques, complete resection of AA lesions can be achieved under colonoscopy, avoiding surgical colectomy at a late CRC stage. Therefore, early detection and timely removal of AA are considered as the effective approaches to prevent CRC development and improving the patient’s quality of life. Recently, the utility of gut microbiota as non-invasive prediction biomarkers for colorectal neoplasia has been explored. For example, *Fusobacterium nucleatum* was reported to be a candidate target to distinguish CRC and adenomas from healthy individuals.^8^ In a 2022 study, Xu et al constructed a prediction model for AA based on microbiota genes, demonstrating excellent performance with an AUC of 0.838.^14^ However, Xu’s diagnostic panel was composed of 277 gene markers, which might limit its generalization capability and application value in clinical practice.

There are several limitations to be addressed. First, the sample size of our study was relatively small, and all the participants were recruited from the Beijing region. In the future, nationwide multi-center trials with large cohorts are needed to validate our findings. Secondly, metagenomic analysis provided the possibility of in-depth screening of diverse microorganisms. The non-bacterial species, such as viruses, fungi, and archaea, accounted for less than 1% of the total microbiota, and yet they act as essential regulators in maintaining gut health. The effect of the sophisticated interplay among different taxonomic species on AA occurrence deserves to be further elucidated. Thirdly, due to the study design, only AA and NC participants were enrolled in the present trial. Next, more pathological types of CRC-associated lesions, including NAA and advanced CRC, are required to explore the dynamic microbial transitions along colorectal tumorigenesis in Chinese populations.

In conclusion, our study revealed altered gut microbiome and functional pathways in AA patients. Compared to normal controls, AA patients displayed a reduced complexity of the microbial co-occurrence network. Furthermore, we generated a novel diagnostic panel, including 3 microbiota biomarkers (*Roseburia inulinivorans*, *Faecalibacterium longum,* and *Bacteroides caccae*), which showed considerable efficiency in AA differentiation. This work may enrich the knowledge of dynamic microbial characteristics along colorectal tumorgenesis and bring new insights into the underlying etiology of AA.

## Figures and Tables

**Table 1.** Baseline Characteristics of AA Patients and NC Participants

Characteristics	AA Group (n = 26)	NC Group (n = 26)	*P*
Age, years, mean ± SD	63.2 ± 8.0	62.4 ± 7.1	.688
Sex, n			1.000
Female	9	9	
Male	17	17	
BMI, kg/m^2^, mean ± SD	25.0 ± 2.9	24.2 ± 2.6	.310
Smoking status, n, %			.368
Active	12 (46.1)	14 (53.8)	
Quit	4 (15.4)	1 (3.9)	
Never	10 (38.5)	11 (42.3)	
Pathological classification (most malignant adenoma), n, %			
Tubular adenoma ≥ 10 mm	5 (19.2)	NA	NA
Villous histology	6 (23.1)	NA	NA
High-grade dysplasia	15 (57.7)	NA	NA

AA, advanced adenoma; BMI, body mass index; NC, normal control.

## Data Availability

The data that support the findings of this study are available on request from the corresponding author.
