# Supplementary material for: Alterations of Gut Microbiome in Patients with Colorectal Advanced Adenoma by Metagenomic Analyses
Source: Turk J Gastroenterol. 2024 Nov 1;35(11):859–68. doi: 10.5152/tjg.2024.24294 (PMC11562533; doi:10.5152/tjg.2024.24294)
Supplement: Supplementary Material [file supplementary_material.pdf]

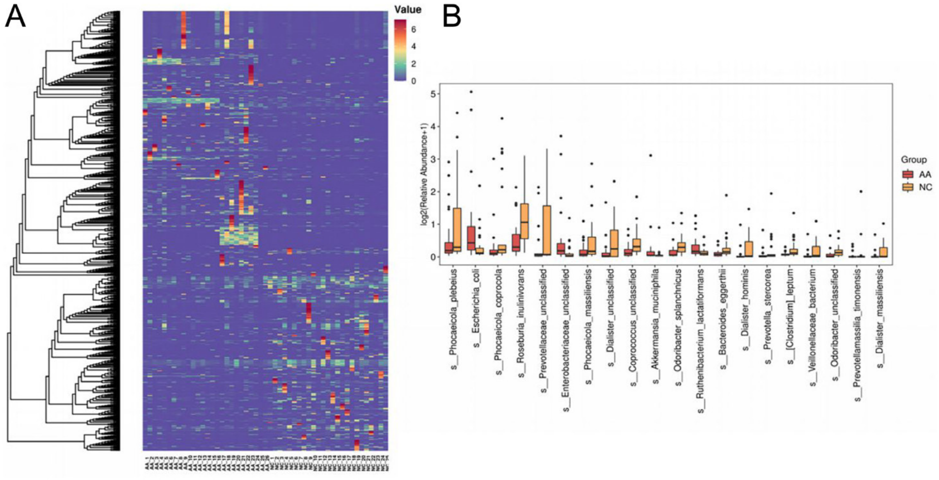

Supplementary Figure 1. Microbial profiles of AA patients and NC individuals at the species level. (A) Heatmap showed a clear difference of microbial composition between two groups. (B) Differentially expressed microbiota species (top 20) in AA.
